# Supplementary material for: Phenome-wide analysis of Taiwan Biobank reveals novel glycemia-related loci and genetic risks for diabetes
Source: Commun Biol. 2022 Nov 3;5:1175. doi: 10.1038/s42003-022-04168-0 (PMC9633758; doi:10.1038/s42003-022-04168-0)
Supplement: Supplementary file 2 — Supplementary Information [file 42003_2022_4168_MOESM2_ESM.pdf]

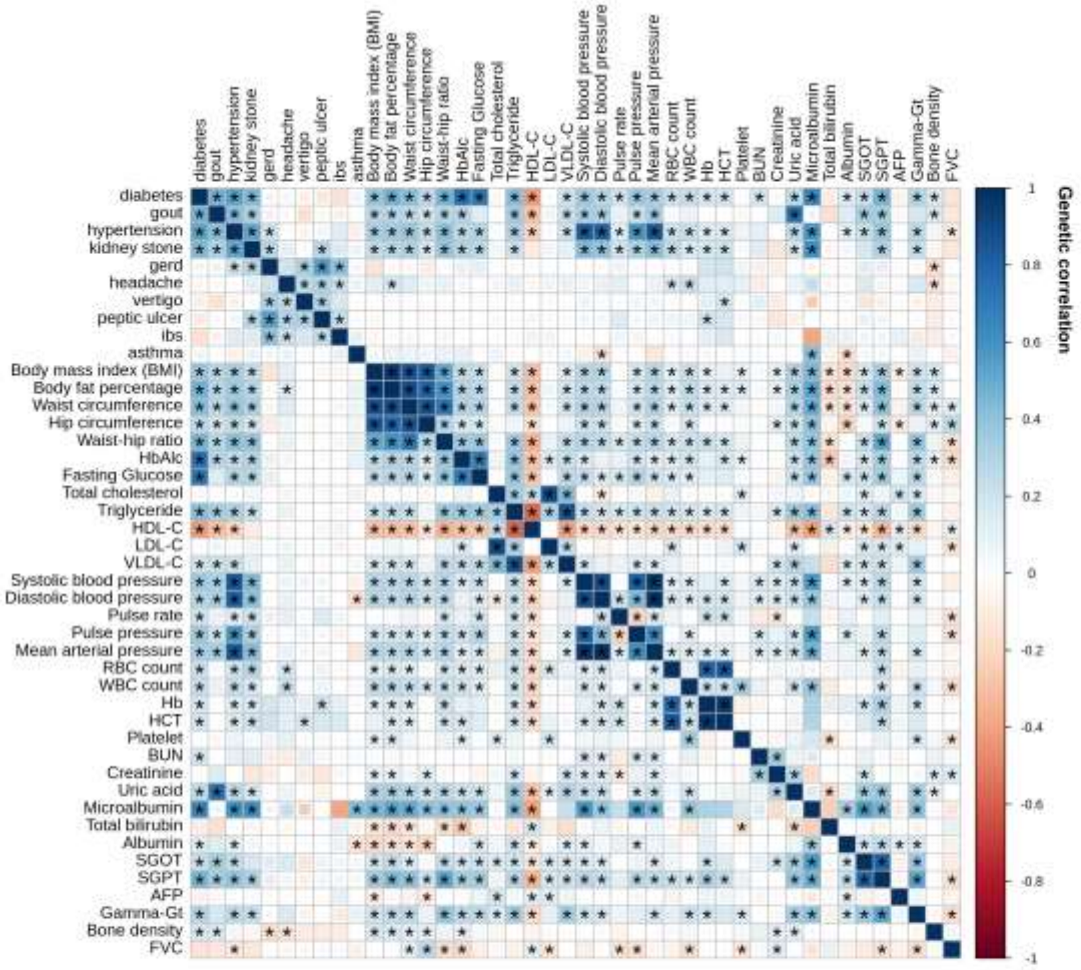


**Supplementary Fig. 1 Pairwise genetic correlation among 44 traits present in TWB.** Pairwise genetic correlations (n = 946 pairs) were estimated using the bivariate LD score. Positive and negative correlation are colored in blue and red respectively. The intensity of correlation is indicated by the color saturation. The FDR is calculated by the Benjamini–Hochberg method. Size of the color block represents the FDR of each correlation, and significant correlations (FDR ≤ 0.05) are indicated by asterisks. Pairwise genetic correlation was estimated with summary statistics calculated using baseline model (age + sex + first 10 PCs).

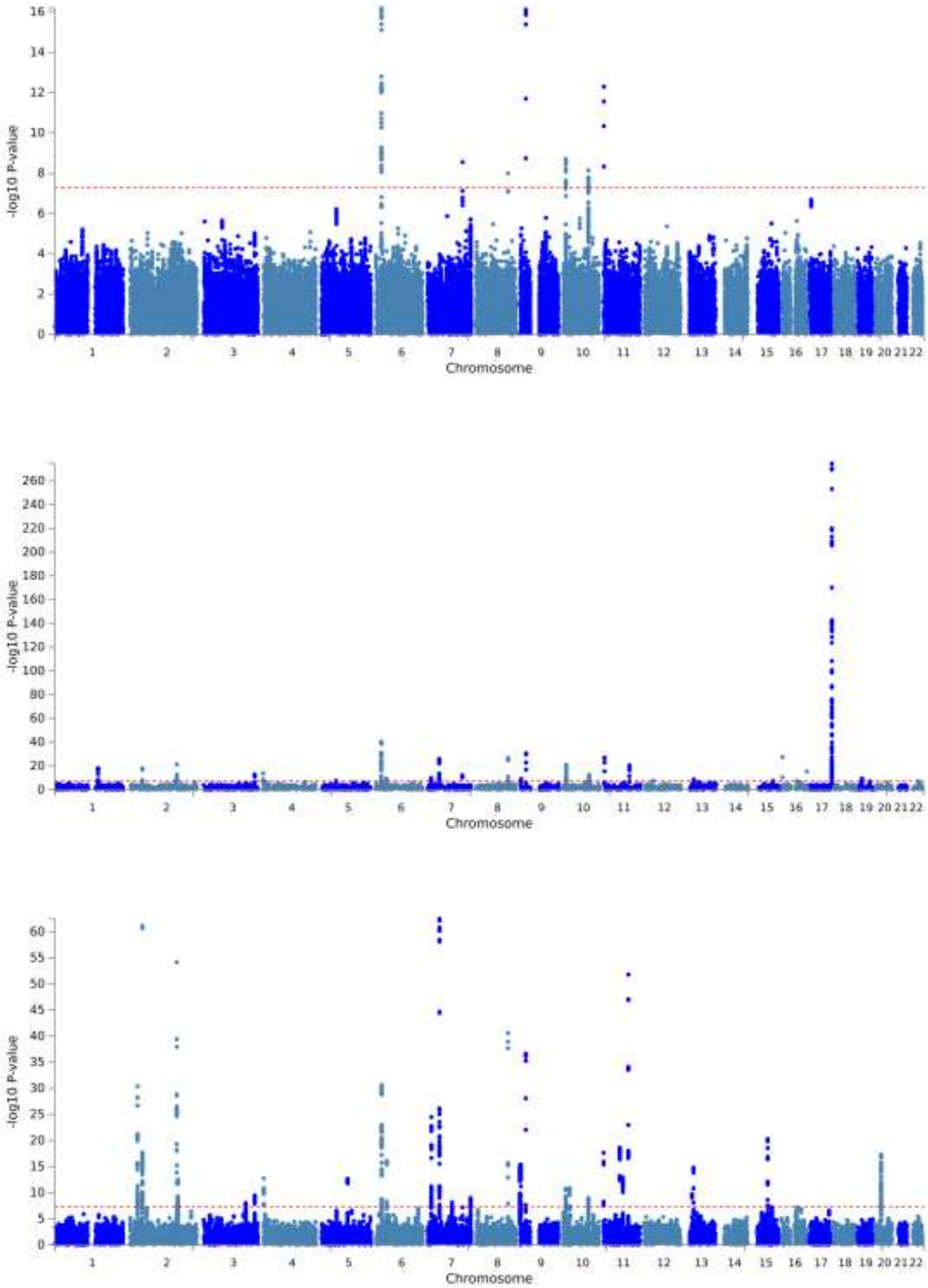


**Supplementary Fig. 2 Manhattan plot for GWAS of T2D (top), $HbA_{1c}$ (middle) and fasting glucose (bottom).** Red line indicates genome-wide significant association at p-value < $5x10^{-8}$.

| | T2D | HbA1c | Fasting Glucose |
|---|---|---|---|
| #Genomic risk loci | 7 | 26 | 29 |
| #lead SNPs | 7 | 40 | 41 |
| #Ind. Sig. SNPs | 9 | 88 | 106 |
| #candidate SNPs | 302 | 2771 | 4142 |
| #candidate GWAS tagged SNPs | 168 | 1219 | 2075 |
| #mapped genes | 65 | 440 | 615 |

**Supplementary Table 1.** Summary of SNPs and mapped genes

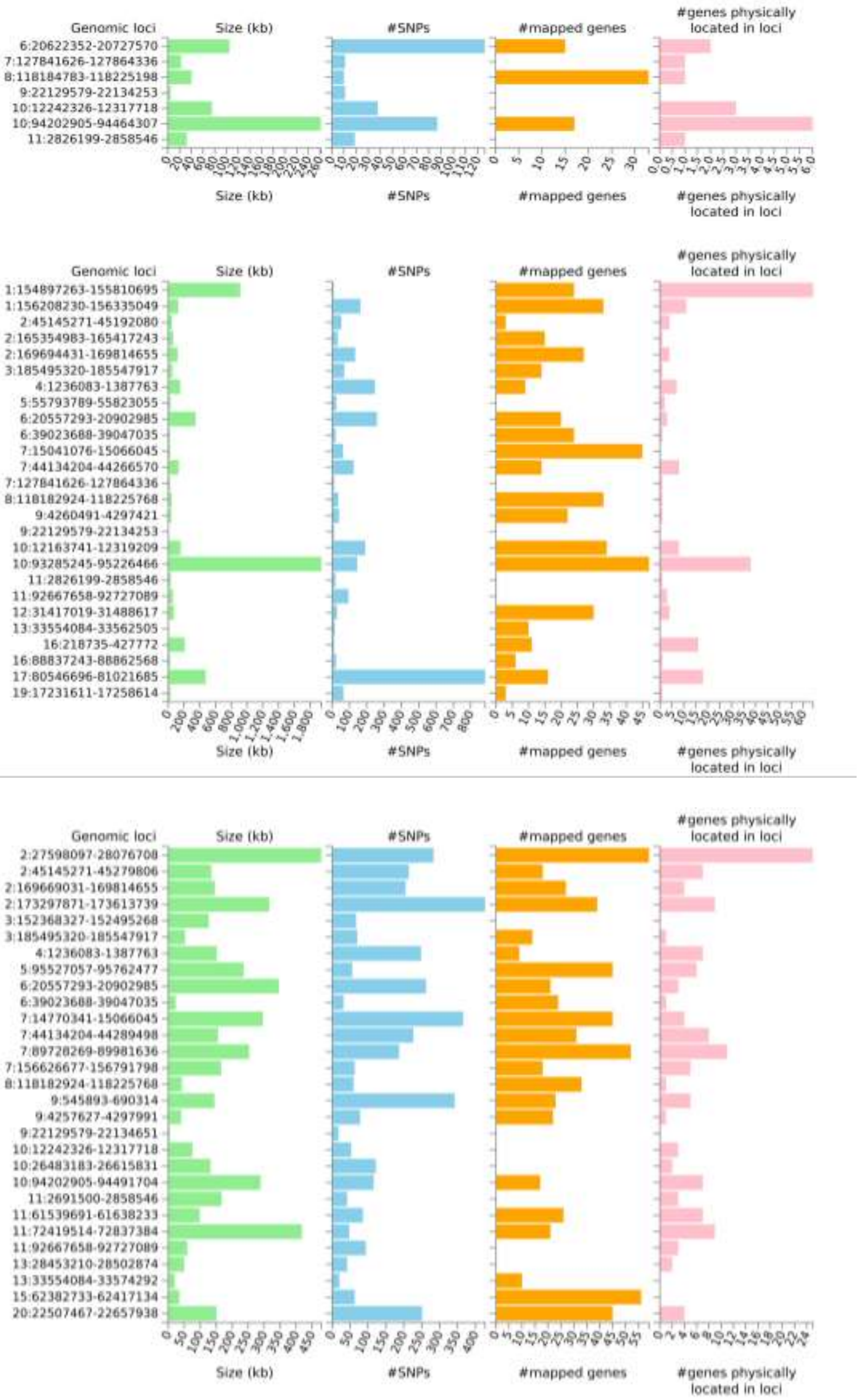


**Supplementary Fig. 3 Summary of FUMA SNP2GENE** for GWAS of T2D (top), $HbA_{1c}$

(middle) and fasting glucose (bottom).

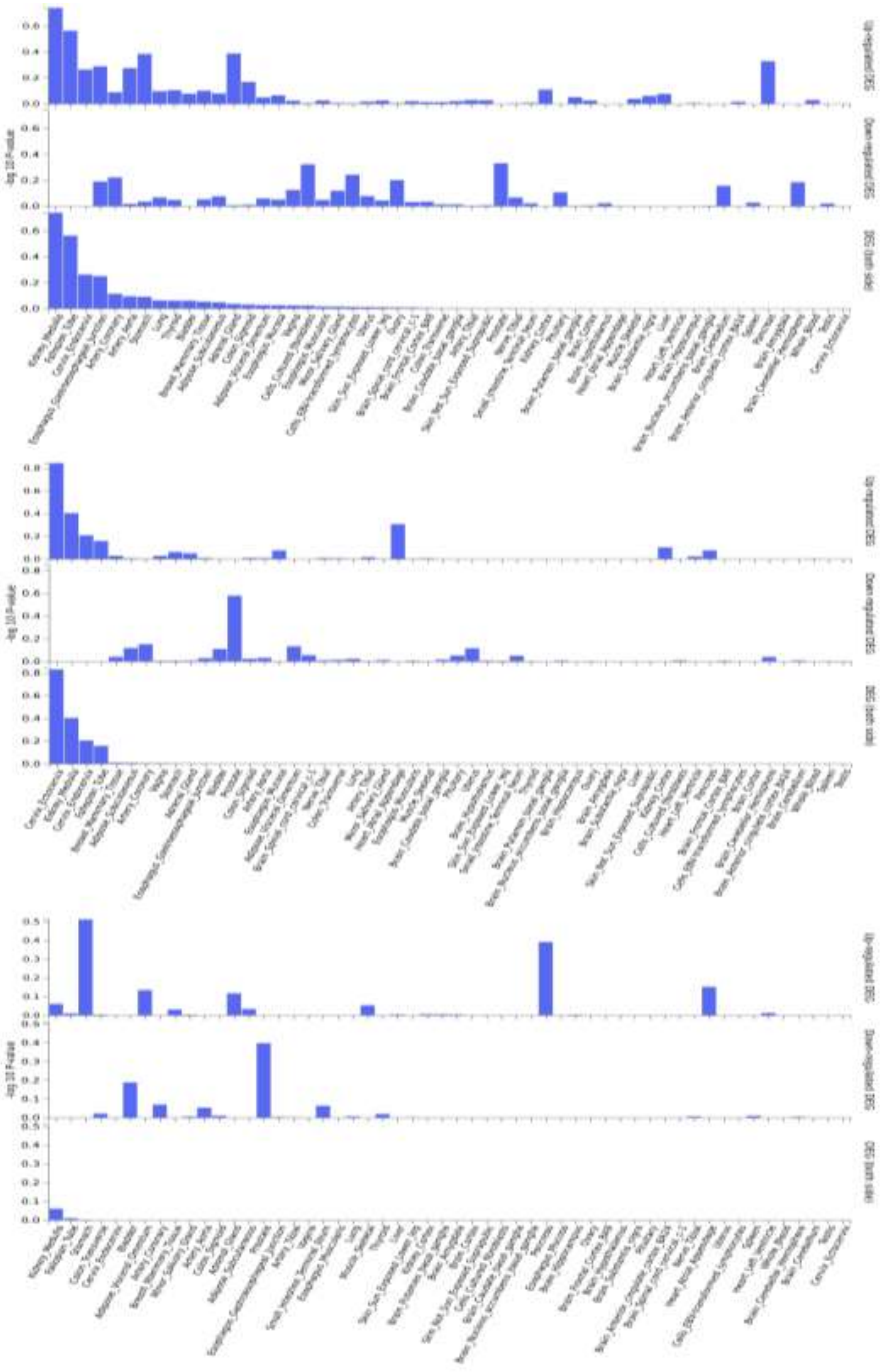

**Supplementary Fig. 4 Differentially expressed genes of GTEx v8 54 tissue types**

with genes mapped from GWAS summary statistics of T2D (top), $HbA_{1c}$ (middle) and fasting glucose (bottom).

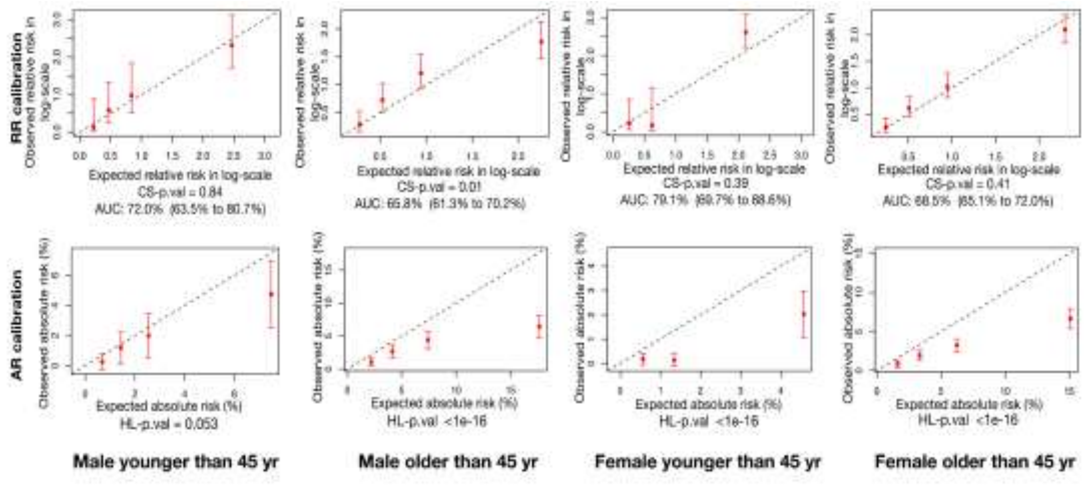


**Supplementary Fig. 5 Model calibration and discrimination for males aged younger or older than 45 years and for female aged younger or older than 45 years.** Model calibration of relative and absolute risk were assessed by chi-square (CS) and Hosmer-Lemeshow (HL) goodness of fit tests, respectively, and p.val is the p-value for the corresponding test. Model discrimination was assessed by AUC = area under the curve and its 95% confidence interval is presented in (). The error bar in red represents the 95% confidence interval for RR and AR respectively [30].

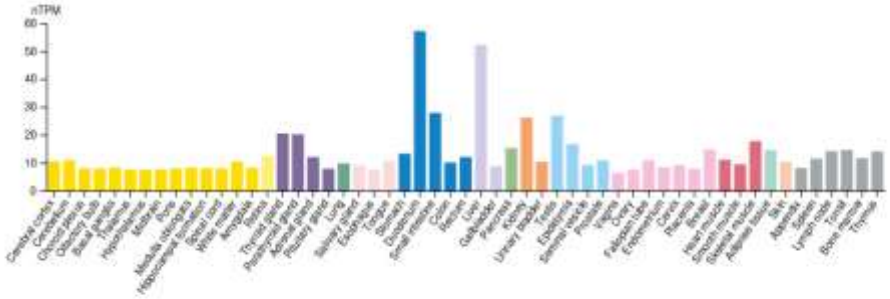


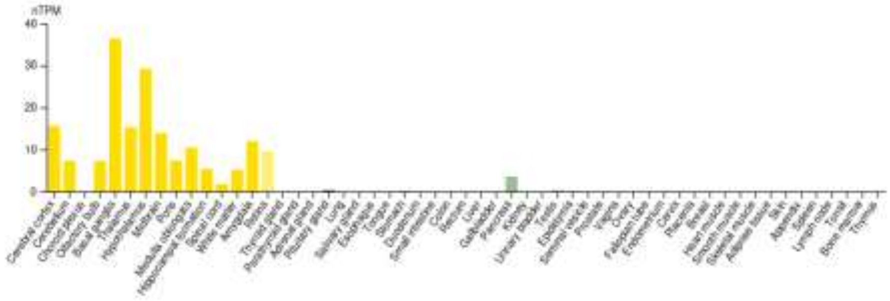


**Supplementary Fig. 6 Consensus transcript expression levels** for *HACL1* (top) and *GAD2* (bottom) in 55 human tissues based on transcriptomics data from the two sources HPA and GTEx generated from Protein Atlas.

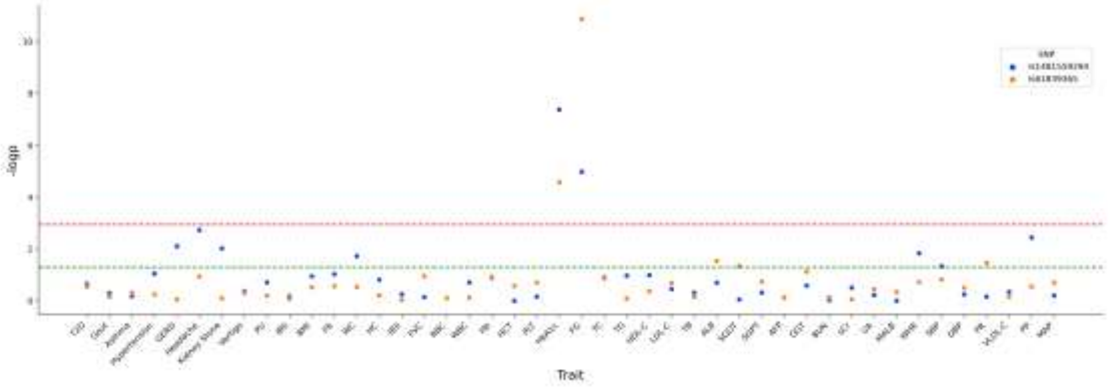


**Supplementary Fig. 7 PheWAS plot** of rs61839365 (*HACL1*) and rs61839365 (*GAD2*) with red line indicating association at p<0.05/44 and green line indicating association at p<0.05.

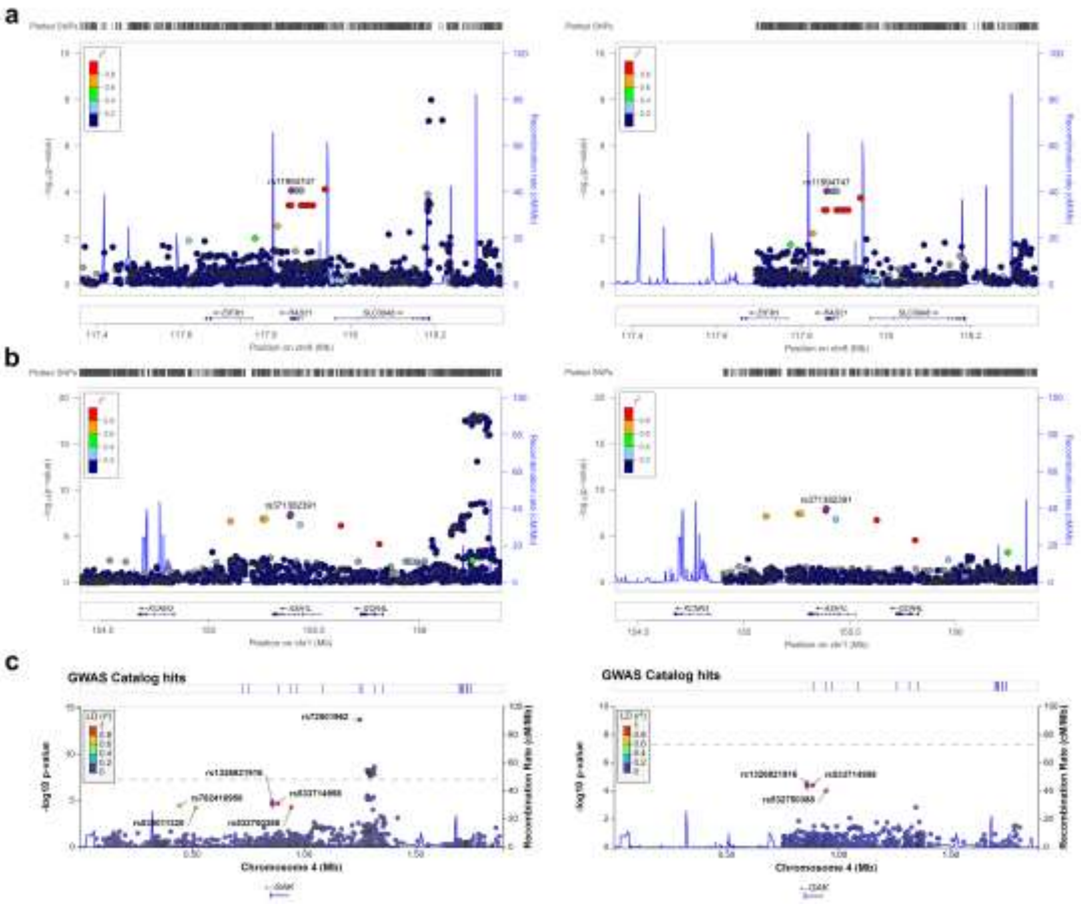


**Supplementary Fig. 8 Regional plots before (left) and after (right) a conditional analysis.** a *RAD21* gene (T2D); b *ASH1L* gene ($HbA_{1c}$ ) and c *GAK* gen (fasting glucose).

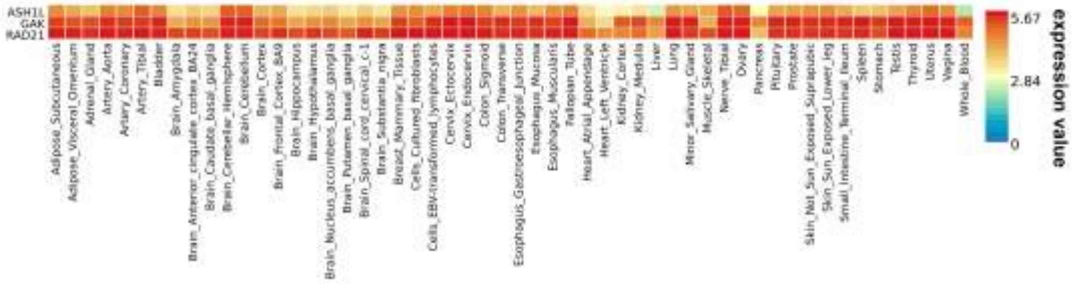


**Supplementary Fig. 9 Averaged gene expression heatmap of *ASH1L*, *GAK* and *RAD21* from GTEx v8 54 tissue types dataset following winsorization at 50 and log 2 transformation with pseudocount 1.** Cells filled in red indicates higher expression compared to cells filled in blue.
