# Supplementary material for: Phenome-wide analysis of Taiwan Biobank reveals novel glycemia-related loci and genetic risks for diabetes
Source: Commun Biol. 2022 Nov 3;5:1175. doi: 10.1038/s42003-022-04168-0 (PMC9633758; doi:10.1038/s42003-022-04168-0)
Supplement: Supplementary file 3 — Description of Additional Supplementary Files [file 42003_2022_4168_MOESM3_ESM.pdf]

Description of Additional Supplementary Files

File name: Supplementary Data 1

Description: Overview of the Taiwan Biobank phenome-wide association study of 44 traits.

File name: Supplementary Data 2

Description: Demographics of study population.

File name: Supplementary Data 3

Description: Significantly associated loci.

File name: Supplementary Data 4

Description: Significant pleiotropic loci.

File name: Supplementary Data 5

Description: Additional independent loci.

File name: Supplementary Data 6

Description: Pairwise genetic correlation.

File name: Supplementary Data 7

Description: Reference(s) of significant genetic correlations.

File name: Supplementary Data 8

Description: Association SNPs for glycemic traits.

File name: Supplementary Data 9

Description: Instrumental variables used in MR analyses.

File name: Supplementary Data 10

Description: Two-sample MR analyses of HbA1c exposure in TWB participants.

File name: Supplementary Data 11

Description: Two-sample MR analyses of FG exposure in TWB participants.

File name: Supplementary Data 12

Description: Functional annotation of SNPs for T2D.

File name: Supplementary Data 13

Description: Functional annotation of SNPs for HbA1c.

File name: Supplementary Data 14

Description: Functional annotation of SNPs for FG.

File name: Supplementary Data 15

Description: 10-year absolute risk of type-2 diabetes in the Taiwanese population aged 30 to 50.
